# Half a century of quantitative cultural evolution

**DOI:** 10.1073/pnas.2418106121

**Published:** 2024-11-18

**Authors:** Laurel Fogarty, Anne Kandler, Nicole Creanza, Marcus W. Feldman

**Affiliations:** ^a^Department of Human Behavior, Ecology and Culture, Max Planck Institute for Evolutionary Anthropology, Leipzig 04103, Germany; ^b^Department of Biological Sciences, Vanderbilt University, Nashville, TN 37235; ^c^Department of Biology, Stanford University, Stanford, CA 94305

## A Brief History of Quantitative Cultural Evolution

Culture is a powerful force that touches all aspects of human life, from health and reproduction to food and clothing technology, in every known human population around the world. Human cultural traits are passed between generations and within cohorts and families, resulting in vast legacies of complex institutions, norms, and technologies without which human life would be unrecognizable. A model of human evolution that does not include at least a cursory account of human cultural change can only produce an incomplete and fragmentary picture of our evolutionary history.

Cultural change shares important features with biological evolution. The correspondence between biological evolution and cultural change, although not exact, has been noted throughout the history and development of evolutionary thought. Linguist August Schleicher suggested that the basic features of Darwin’s “great struggle for survival” (1) could be applied to the rise and fall of one very particular human cultural trait—language—with “little or no alteration” (2). In 1871, with the publication of The Descent of Man, Darwin (3) directly pointed to what he saw as a “curious parallel” between the ways in which species change over time and the ways in which human languages do the same.

Following the rediscovery of Mendel’s work, Darwin’s ideas about natural selection were crystallized into a formal mathematical theory of evolution by natural selection by Fisher, Wright, Haldane, and others. The “modern synthesis” between evolutionary theory and Mendelian inheritance (4) offered to explain biological evolution almost completely (5).

This gene-based evolutionary theory also held great promise for explaining human evolution in particular. However, building on early eugenics, insights from genetics were increasingly misused to justify and support racist ideas, particularly regarding the heritability of complex phenotypes like IQ (e.g. refs. 6 and 7). These ideas spread from academia into political and social spheres. Many geneticists objected to this use of genetics to justify not just racist ideas, but racist policies and actions (8, 9). Instead, they suggested, many estimates of the heritability of complex human behavioral traits were influenced in myriad ways by the environment (10, 11) and, significantly, by culture (12–14).

Public pronouncements about the supposed genetic determination of intelligence and other behavioral phenotypes drove Stanford geneticists Luigi Luca Cavalli-Sforza and Marcus W. Feldman to formalize, for the first time, the cultural components of phenotype and to begin the development of a theory of cultural evolution alongside the burgeoning theory of genetic evolution. What emerged was the first mathematical formalization of the “curious parallel” between genes and culture noted by Darwin and others more than a century earlier (3). In this formalization, cultural traits were treated as entities that could be transmitted, not just from parents to offspring in strict Mendelian fashion (as genes are most often transmitted), but also through other channels or modes of transmission, such as from peer to peer or from specially chosen role models to naïve individuals.

The misunderstanding and misuse of evolutionary concepts that sparked the development of cultural evolution continue in academic circles and beyond today. In their contribution to this special feature, Lala and Feldman (15) focus on the modern face of scientific racism, and show how a deep understanding of cultural evolution, and how genes and culture interact through gene–culture coevolution, can counter naïve, incorrect, and harmful interpretations of human genetic data. The paper makes clear that a complete account of human evolution encompassing all of our genetic similarities and differences must account for the profound effect of human cultural traits on human phenotypes.

The development of the first mathematical models of cultural evolution also marked the beginning of a 40-y collaboration between Cavalli-Sforza and Feldman. The contribution of Cavalli-Sforza’s biographers (16) Stone and Lurquin to this special feature (17) elucidates the motivations and the intellectual roots of Cavalli-Sforza’s contributions to cultural evolution in the context of his full career—ranging from the study of bacterial genetics, human language, anthropological fieldwork, human genetics, to cultural evolution. Stone and Lurquin (17) trace the collaboration at the heart of the field’s origins, which drew genetics and evolutionary theory together in a unique way, strongly influencing the trajectory of the field over the subsequent 50 y.

The first models published by Cavalli-Sforza and Feldman (12, 13) suggested that a full theory of “cultural evolution” could help to explain human cultural diversity, inheritance, and biological evolution—just as genetic evolutionary theory had done for the diversity of biological species. They showed that the inheritance of cultural traits could have a significant effect on measures of human phenotypes and their familial relationships, with serious consequences for the study of human genetics and heritability in general. Quantitative cultural evolution rapidly branched out from its origins in the field of human population genetics, to anthropology, archaeology, and beyond.

This PNAS Special Feature celebrates the 50th anniversary of the publication of the two landmark models of cultural evolution, both published in 1973, which marked the beginning of the field of quantitative cultural evolution. The articles contained in this special feature map the rapid expansion of the field of quantitative cultural evolution over the last 50 y and illustrate the insights and progress that the theory of cultural evolution has made, and continues to make, in the wide array of academic fields that study human and animal culture.

## The First Quantitative Models of Cultural Evolution: Cavalli-Sforza and Feldman (12, 13)

Despite the similarities between cultural and genetic evolution recognized by Darwin and others, the cultural transmission of phenotypically relevant information may have dynamics quite different from genetic transmission. For example, while human genes are passed from parents to offspring, cultural traits—defined broadly as traits that are transmitted from one individual to another by learning—might be passed from any number of cultural models (including parents) to a naïve individual.

Cavalli-Sforza and Feldman (12) explicitly included alternative transmission pathways for cultural information in a model of uniparental transmission that included an oblique “group effect.” The first cultural transmission pathways to be modeled involved vertical transmission, namely transmission of cultural information from genetic parents to their offspring, and random oblique transmission, namely transmission from randomly chosen, and possibly unrelated, group members to naïve individuals. With a simple description of a genetically and culturally determined phenotype, it was possible to follow how the population phenotypic mean and variance changed over time and to what extent that pattern of change would be altered if the phenotype were more vertically transmitted or obliquely transmitted. The authors focused on trait variation, which they showed depended on this balance between vertical and oblique transmission and, in elaborations of the model, on the number of cultural parents.

The second model published by Cavalli-Sforza and Feldman (13) detailed how vertical cultural and genetic transmission operate together and how these can change the correlation between relatives in key phenotypic characters. Here, the oblique group effect, which was related to the population’s mean trait value, was replaced with an explicit model of oblique transmission operationalized as transmission from randomly chosen adoptive parents.

In this model, vertically transmitted cultural and genetic sources of information were pulled apart and modeled as distinct processes. This model formalized the idea of phenotypic inheritance by allowing the phenotype of a new individual to result from a particular mating and to go through a process of development determined by offspring genotype. The aim was to use the model to investigate family resemblances: How similar, for example, should we expect a parent and their offspring to be under the influence of both genes and transmitted culture? How similar should we expect siblings to be to one another? And how much of that similarity in complex human phenotypes is attributable to genes and how much to culture?

## Further Theoretical Development

From the first models of cultural transmission in 1973 and to the present day, there has been a tight connection between cultural evolutionary theory and the theory of population genetics. The foundations of much modern cultural evolutionary theory can be traced directly to models and methods from population genetics, which formed a baseline from which a theory of the transmission and evolution of cultural traits could be developed. The relationship between the two disciplines runs deep, and, drawing this out in their article, Wakano and Aoki (this special feature, 18) focus on two particularly illustrative points of interaction. First, they revisit a long-standing question—what are the origins of the near universal incest taboos in human societies, and how are they maintained? Here, they demonstrate that careful accounting for transmission dynamics and tracking similarity between relatives, both fundamental elements of population genetic models, can be applied to a case of cultural transmission relevant to human health and well-being. Using population genetic methods to describe cultural evolution offers clear paths toward a deeper understanding of how human social structure aligns with genetic structure, and how genetically relevant cultural traits can spread and be maintained. Second, they show how modern coalescent theory and “backward time” methods can be leveraged to better understand cultural datasets and the deep history of human culture. These two examples together illustrate how the close relationship between the fields of population genetic theory and cultural evolutionary theory has been pivotal to the development of the field of cultural evolution and also that this relationship continues to develop, contributing new methods to address generalized transmission as well as novel perspectives on cultural datasets.

A crucial factor driving the development of cultural evolution, and its growth as a field, was the involvement of anthropologist Robert Boyd and human ecologist Peter J. Richerson. Beginning in 1978 (19), Richerson and Boyd published a series of theoretical and conceptual models of cultural evolution. Their early work developed two concepts in particular—guided variation and biased cultural transmission—and added a new psychological component to existing models (20). Their popular book “Culture and the Evolutionary Process,” published in 1985, has become an accessible and frequently cited source for students of cultural evolution. In some of their most impactful work, Boyd and Richerson developed theory and conceptual frameworks that extended cultural evolutionary models by allowing cultural transmission to be frequency-dependent, e.g., via conformity bias. They also focused on the role of cultural evolution in the evolution of large-scale human cooperation (21–25). Thus, the work of Boyd and Richerson has built an important branch of cultural evolution as it developed through the 1980s and 1990s, to the present day. In their perspective article, Boyd and Richerson (26) describe the intellectual roots of their interest in cultural evolution—not in population genetics—but at the intersection of social evolution and anthropology. They address the controversies that the field has faced and offer their perspective on the future of cultural evolution.

Two early papers from the collaboration between Cavalli-Sforza and Feldman (13, 27) represented the first mathematical formulations of a gene–culture coevolutionary theory—examining the inheritance of genes alongside the transmission of cultural traits and exploring how these might interact (28–31). The mathematical development of gene–culture coevolutionary theory has lagged behind the development of genetic theory even where that theory has been applied to human genetic analyses. In this special feature, Fogarty and Otto (32) develop a model describing the signatures that cultural processes might leave in genetic data. To this end, they operationalize two forms of cultural transmission that can lead to the formation and maintenance of stable associations between cultural traits and genetic alleles in a gene–culture coevolutionary framework (20, 33). Focusing on gene–culture hitchhiking and cultural interference in genetic selective sweeps they demonstrate the effect that culture can have on shaping genetic diversity. This modeling framework, and future developments of it, will allow for predictions of the potential strength of cultural effects on genomic data, identifying situations where such data cannot be adequately interpreted without first understanding gene–culture interactions.

## Spread to Other Disciplines and Applications

The theory of cultural evolution that was developed over the course of the last fifty years has spread to many other disciplines where understanding human culture is of particular importance. One of the clearest links to other fields, and one of the earliest to be forged, was the link to anthropology. In 1986, after years of researching the lives of the Aka people in Central African Republic together, anthropologist Barry S. Hewlett and L. L. Cavalli-Sforza conducted one of the first field studies aimed at quantifying cultural transmission and testing the models of Cavalli-Sforza and Feldman. Until this study, most anthropological work on cultural spread had focused on socialization and the spread of values, attitudes, or personality traits (34). Few studies had aimed to investigate, statistically, how practical skills such as artifact production or foraging strategies were transmitted. However, it was exactly these kinds of traits, often learned from specific known sources, that were appropriate for studying cultural transmission and evolution in Cavalli-Sforza and Feldman’s framework. Hewlett and Cavalli-Sforza (34) described and empirically tested a collection of “modes” of cultural transmission through which, they suggested, hunter-gatherer children might learn important subsistence and survival traits. They estimated that vertical cultural transmission was extremely important in this population [a finding that was later replicated and refined in other studies (e.g. refs. 35–38)] and that the cultural transmission mechanisms used to spread different types of information could be linked to population-level patterns of variation in cultural traits. Hewlett et al. (this special feature, 39) revisit and extend this landmark work, empirically investigating five modes of transmission, adding nuance to our understanding of horizontal, oblique, and vertical cultural transmission. Hewlett et al. (39) show how some of the most salient and important features of hunter-gatherer culture in the Congo Basin foragers, among them extensive sharing and egalitarian social structures, are maintained across generations through cultural transmission mechanisms specific to particular traits. These analyses emphasize the role that cultural evolutionary theory has played in driving forward our understanding of human culture, how it evolves, and how it is maintained.

Cavalli-Sforza saw another role for the scientific study of human culture. Culture, he reasoned, might hold important information about the evolutionary roots of our species (40). This line of research builds on Darwin’s prediction in On the Origin of Species that “a perfect pedigree of mankind …would afford the best classification of the various languages now spoken throughout the world” (1)—in other words, that a phylogeny of human populations should closely match a linguistic phylogeny. Cavalli-Sforza’s early research on phylogenetics led to some of the first genetic phylogenies of human populations (e.g. ref. 41). Beginning in 1988, Cavalli-Sforza et al. (42) attempted to align these genetic phylogenies with those of language families. They showed that the genealogical and migratory history of our species can be told by the distribution of genes around the world—and by the distribution of languages (40, 43). More recent analyses with much larger datasets have supported the broad concordance between genetic and cultural data, which retain signatures of human demographic history (44–46). The correspondence between genes and languages in these analyses demonstrated many things, among them, that cultural traits and how they are transmitted might create and maintain historically important population boundaries. These cultural distinctions, in turn, might influence the formation of families and the transmission of genes. This form of gene–culture coevolution, and detecting it, is the focus of Pichkar et al. [this special feature (47)]. Pichkar et al. increase the resolution of such analyses and suggest that if a child is more likely to learn language from their mother compared to their father, language distribution data should more closely align with the distribution of maternally biased genetic markers (X chromosomes and mitochondrial genes) than with autosomal genes passed on by both mothers and fathers. Again showing the power of culture to shape genetic diversity, their analysis suggests that such a relationship between mtDNA and language tends to exist where other cultural traits such as female-based residence patterns can facilitate it.

Language, as a very persistent but ever-changing part of human culture, has also been the subject of many attempts to construct “cultural phylogenies,” in other words, to reconstruct phylogenetic relationships between cultural traits that may share ancestry (e.g. see refs. 48 and 49). Linking cultural microevolution to these kinds of evolutionary trees has led to the publication of cultural phylogenies based on a variety of other cultural traits (50–53). Although much basic theory remains to be done in this area (54–57), the idea that such phylogenetic trees can be made is an intuitive one that holds great promise for new methods of analyzing our cultural past.

Anthropological and linguistic data pertaining to cultural diversity, transmission biases, and human migration, although collected under challenging conditions and with great effort, are relatively abundant compared to archaeological data on similar topics. Archaeological data on tools and their use by ancient human populations suffer from several additional challenges including taphonomic and preservation issues, comparatively poor temporal resolution, and unavoidable time averaging. Archaeologist Stephen Shennan was instrumental in bringing the framework of cultural evolutionary theory into the field of archaeology (58–61) see also (e.g. refs. 62 and 63). This work suggested that the cultural transmission of human artifacts, even in the distant past, could be described and understood and that the archaeological record, despite its sparsity, likely contained identifiable signatures of cultural transmission mechanisms accumulated over vast timescales. Shennan’s work, among others, has shown that examining human culture over the timespan of human evolutionary history requires a careful analysis of the potential interactions between genetic and cultural evolution. Large-scale changes to human lifeways, such as the advent and spread of agriculture (64), have important genetic consequences. In this special feature, Carrignon et al. (65) examine the dynamics of spread of a beneficial cultural trait (such as agriculture) and the associated spread of hitchhiking neutral cultural traits, which might be more easily identified in archaeological assemblages. They particularly focus on how cultural norms, such as marriage and migration practices, and modes of socialization may influence the spread of agriculture and the hitchhiking of other traits, for example, pottery designs. They conclude that observable patterns in the co-occurrence of cultural traits might help archaeologists to learn something about modes of cultural transmission, sex biases in learning, or even migration patterns in historical populations, from careful interpretation of archaeological assemblages (see also ref. 32).

The sparse data with which archaeologists typically work contrast sharply with the “era of big data” in which many cultural evolution researchers now operate (e.g. refs. 66–68). As ready access to large-scale data detailing the transmission of information, for example between users of social media websites, steadily increases, the field of cultural evolution has been faced with a new and quite different challenge: large-scale data analysis. How can large databases that combine information from numerous anthropological field sites, historical sources, or websites be properly analyzed? The scientific rigor of cultural evolutionary data analysis has become an urgent area of research and development (69). While the use of electrophoresis and later genomics pushed population genetics forward as an inferential science (70, 71), leading to the rapid development of statistical and theoretical frameworks, the same is not yet the case for the field of cultural evolution. In this special feature, Deffner et al. (72) describe a toolbox of statistical methods and develop workflows to create a stronger link between cultural evolutionary theory and large cultural datasets. Using simulated data, they demonstrate a workflow from study design, to statistical analysis and inference. Such clear, careful, and well-documented applications of cultural evolutionary theory to cultural data collection and analysis have the potential to hugely improve our interpretation of cultural datasets.

Alongside its role in scholarly contexts, the theory of cultural transmission and evolution has also been applied to a number of urgent social issues. In cases where harmful cultural norms are strongly maintained, leading to serious social problems (73), or where the transmission of deleterious behaviors or beliefs [e.g., vaccine hesitancy (e.g., refs. 74–76)] might have negative effects on public health, understanding how cultural traits are transmitted and maintained in populations is of great practical importance. In some such contexts, it might be possible to use cultural evolutionary theory to predict the efficacy of a range of potential interventions (77) or to generate a deeper understanding of the interaction between human culture and human health in general (78). In this special feature, Pooladvanda et al. (79) use cultural evolutionary theory to examine the link between novel human cultural traits and the emergence of new infectious diseases. Their work, and future developments based on it, have important applications, particularly as we struggle to understand how changes in human behavior, culture, and living conditions may have led to the recent deadly covid-19 pandemic pandemic in 2020. Pooladvanda et al. investigate the rate of occurrence of often-unintended maladaptive consequences following the spread of seemingly positive cultural traits, and suggest that the high rate of social learning in this context is a double-edged sword. On one hand, rapid social learning makes maladaptive outcomes more likely, and on the other, it provides a possible solution—the rapid spread of safer cultural practices. This study demonstrates that a detailed understanding of human cultural evolution—adding to more psychology-focused work on human behavior in the context of infectious disease (80–82)—is likely to become increasingly important as more of the pathogens that humans face result from our changing interactions with the natural world, mediated, ultimately, by our cultural practices.

Culture and cultural transmission are not unique to humans, and many nonhuman animals also have culturally transmitted traditions (e.g. refs. 83–87). There is, therefore, a natural link between animal social learning and human cultural evolution. Many models of nonhuman animal social learning originated in the field of psychology and from models of cultural transmission (29). Empirical work on animal social learning has a rich history in psychology, where an interest in how, when, and what animals learn has persisted for at least a century (29). However, a large amount of this work has focused on facets of the cognition underpinning learning in humans, paying less attention to the population consequences of social learning in nonhuman animals—in other words, the potential for animal culture. Kevin Lala and colleagues (29, 88) pioneered the use of models of cultural evolutionary theory to investigate the population-level consequences of social learning biases for the evolution of animal social learning and animal cultures. They modeled the evolution of animal social learning, mapping out the circumstances under which we might expect to see social learning, innovation, and population-wide animal traditions emerge (e.g. refs. 88–90). A major contribution from the mathematical formalization of animal social learning was to guide population-level empirical research. Now, much of the empirical research on animal social learning is developed in close association with population-level theoretical frameworks (e.g. refs. 91–93). One such theoretically focused empiricist bridging this gap is Rachel Kendal (e.g., refs. 94 and 95). Here, Galheigo Coelho et al. (96) report on an example of this close interplay between theory, empirical observations of wild animal social interactions, and sophisticated statistical analysis. They studied bearded capuchins—a species of monkey with an unusually large toolkit thought to be maintained by social learning. Drawing on models of social tolerance, they demonstrated cultural transmission in bearded capuchins for the first time, and described transmission mechanisms that were likely to underlie the behavioral patterns they found. The paper demonstrates the close interaction between cultural evolutionary theory, empirical study design, and statistical modeling now used by animal behavior researchers interested in social learning.

As the work on animal social learning demonstrates, models of cultural evolution can make several testable predictions about cultural transmission mechanisms, the circumstances under which they might be relevant, and their population-level consequences. In humans, early empirical studies of cultural transmission focused on observational data from hunter-gatherer populations (34) or on surveys completed by various undergraduate populations (35). However, the data needed to validate many theoretical predictions are difficult to obtain in an observational setting. Fine-grained data detailing who is learning from whom and what strategies are employed under particular circumstances are extremely difficult to obtain (but see, e.g., refs. 97–100). Adding to observational data, much can be achieved in the controlled environment of a laboratory.

Some of the first laboratory tests of cultural evolution developed simulation models that could be tested directly by experiments on human subjects in the lab (e.g. refs. 101 and 102). “Transmission chain” methods, pioneered in the field of psychology by Bartlett in 1932 (103), have been used to study cultural transmission in the lab (e.g., refs. 104–108), adding to other methods based on laboratory microsocieties (109, 110). Such methods have been widely applied and have generated insights into the dynamics of cumulative cultural evolution (e.g., refs. 107 and 108), language evolution (e.g., refs. 111–113), population size effects (114), information requirements (115), and the transmission of tool-using technology (116). Alongside other laboratory-focused researchers, such as Maxime Derex and Christine Caldwell, psychologist Alex Mesoudi has brought innovative tests of cultural evolutionary theory into the lab (106, 117) and has driven many synthetic and conceptual advances in cultural evolution (118–120). In this feature, Derex et al. (121) discuss the history of laboratory-based work on cultural evolution, its criticisms, and its successes. They present a lab experiment comparing the effectiveness of learning in chains, in groups, or alone. This line of work provides important information about how cultural transmission actually proceeds under a variety of controlled conditions and serves to refine and direct further theoretical work.

The papers in this special feature focus on areas of research in which cultural evolutionary theory has had the deepest impact. However, cultural evolutionary concepts, frameworks, and models have had a much wider reach. Cultural evolution has been incorporated into the tool-kits of areas as diverse as human behavioral ecology (e.g., refs. 122–127), conservation studies (e.g., refs. 128 and 129), social psychology (e.g., refs. 130–132), history (e.g., refs. 133–135), economics (e.g., refs. 136–138), and the arts (139, 140).

## The Next 50 y of Cultural Evolutionary Research

“*The basis of culture is the ability to accumulate knowledge, receiving it from previous generations and handing it on to the next…*”—Cavalli-Sforza and Cavalli-Sforza (141)

From the outset, the field of cultural evolution has focused on strong quantitative descriptions of cultural processes (12, 13). This emphasis set a very particular course for cultural evolution research. A trajectory from rigorous mathematical insight to theoretically grounded statistical analysis, a feature of the related field of population genetics, looks possible for cultural evolution, too. The last fifty years have seen rapid improvement along two particularly important axes: computational power and data availability. Both of these have had and will likely continue to have, profound effects on cultural evolution research.

It is clear that culture, and in particular human culture, is a hugely complex and multifaceted phenomenon. To encompass this complexity, simulation modeling has become increasingly important to continued progress in cultural evolutionary theory development. Recent large-scale simulations, which would have been impossible 50 y ago, have, for example, elucidated the selective advantages of different social learning strategies (142); located elusive equilibria in cultural systems (143); investigated the complex effects of memory, innovation, refinement, and conformity on cultural evolution (144–149) and generated predictions about human demographic history (e.g. refs. 61 and 150–152). Increasingly sophisticated simulation models are providing more meaningful descriptions of the most complex aspects of cultural change—for example, by incorporating important facets of cognition or accounting for peculiarities of cultural innovation processes (e.g. refs. 144 and 153).

The development of such models builds bridges between theoretical and empirical studies as statistical methods improve. Generative inference frameworks for cultural evolution (e.g. ref. 154) have the potential to straightforwardly tackle causal questions (for example, “was this archaeological assemblage accumulated by individuals copying one another or through repeated independent innovations?”) by allowing the evaluation of statistical consistency between models of individual-level processes (for example, copying or innovation) and observed population-level patterns (for example, artifact frequencies at a particular archaeological site). The inferential quality of such methods depends in great part on the appropriateness of the generative models used to describe the cultural system. Increased model complexity afforded by improved computational speeds and simulation methods in this context is invaluable.

Gene–culture coevolution is also a particularly rich target for future work as computational resources simultaneously improve both genetic analyses and cultural evolutionary modeling. In this special feature, Fogarty and Otto (32) began to describe what patterns cultural processes might leave in genetic data, which of these might be detectable, and which, if any, of those that are detected might be clearly discernible as cultural effects. Such models are a starting point from which statistical methods to detect the action of culture in the human genome might be developed. The utility of numerical and agent-based simulation is clear—when complex cultural transmission is added to classic models of genetic evolution, analytical solutions are harder to obtain and instead a combination of numerical simulation, agent-based simulation, and mathematical solutions can be used. Similar methods may also resolve patterns of gene–culture geographic covariance, directly linking cultural practices such as residence norms or kinship systems to genetic variation Pichkar et al. (47), or the different effects of demic and cultural diffusion on the cultural evolutionary process (155), see also ref. 65.

Cavalli-Sforza and Feldman (156, p. 340) were clear that the theory they developed required refinement and careful testing against “good data.” At that time, such data were hard to find and detailed cross cultural comparisons on the scale required were challenging. However, alongside the methodological and technological developments described above, recent years have seen datasets on cultural transmission—for example, from social media, pop culture, or gaming websites (e.g. refs. 157–161) and databases collating ethnographic and linguistic data—rapidly expand. For example, digitized databases such as the Human Relations Area Files (eHRAF), D-PLACE (162), Grambank (163), and GeLaTo (45), now collate vast amounts of high-quality cultural and linguistic data, which facilitate cross-cultural comparisons on an unprecedented scale. In combination with principled workflows (72, 154) and appropriate statistical frameworks, testing and refining cultural evolutionary theory and applying it to questions of interest to cultural anthropologists, archaeologists, linguists, or even policy makers has become more tenable.

The quantitative study of cultural evolution began with the aim of preventing shallow analyses of phenotypic data from leading people (either the public or other researchers) to fallacious and dangerous conclusions about how human evolution operates. The project continues fifty years later (Lala and Feldman, this feature). The field is proceeding with increasing sophistication in terms of theory and methods, revealing, as it evolves, new areas of application. Cultural evolution in its current form has now begun to approach the “good theoretical framework” sought by Cavalli-Sforza throughout his career, with which to understand culture (164, p. XX). Cavalli-Sforza (164) and Cavalli-Sforza and Feldman (156) anticipated that advances in genomics and neurophysiology would allow future researchers to understand how culture is transmitted and maintained and to use that knowledge to understand the effects of genes and culture on human phenotypes. However, questions on the exact basis of cultural inheritance remain, as was the case with biological inheritance at the turn of the 20th century. This uncertainty was described by Bateson: “We do not know what is the essential agent in the transmission of parental characters, not even whether it is a material agent or not. Not only is our ignorance complete, but no one has the remotest idea how to set to work on that part of the problem” (165). What we can investigate, and have done successfully for a number of years, is the “collection of facts in great numbers” which Bateson attributed to Mendel in the biological context and suggested as the route toward understanding, ultimately, any natural laws governing inheritance. For cultural evolution, much of this vision is still to be realized, and many questions posed early in the field’s development remain unanswered (156, pp. 340–366). However, theoretical and technical advances have also put many of these answers within reach. New mathematical theory, field data, laboratory experiments, and statistical methods will continue to bring us closer to understanding culture and building a richer and more scientifically grounded description of human evolution.
